# Mechanical Distribution and New Bone Regeneration After Implanting 3D Printed Prostheses for Repairing Metaphyseal Bone Defects: A Finite Element Analysis and Prospective Clinical Study

**DOI:** 10.3389/fbioe.2022.921545

**Published:** 2022-06-03

**Authors:** Bingchuan Liu, Xingcai Li, Weipeng Qiu, Zhongjun Liu, Fang Zhou, Yufeng Zheng, Peng Wen, Yun Tian

**Affiliations:** ^1^ Department of Orthopaedics, Peking University Third Hospital, Beijing, China; ^2^ Engineering Research Center of Bone and Joint Precision Medicine, Ministry of Education, Peking University Third Hospital, Beijing, China; ^3^ School of Materials Science and Engineering, Peking University, Beijing, China; ^4^ Department of Mechanical Engineering, Tsinghua University, Beijing, China

**Keywords:** 3D printing technology, finite element analysis, metaphyseal bone defect, new bone regeneration, clinical study

## Abstract

Critical metaphyseal bone defects caused by nonunion and osteomyelitis are intractable to repair in clinical practice owing to the rigorous demanding of structure and performance. Compared with traditional treatment methods, 3D printing of customized porous titanium alloy prostheses offer feasible and safe opportunities in repairing such bone defects. Yet, so far, no standard guidelines for optimal 3D printed prostheses design and fixation mode have been proposed to further promote prosthesis stability as well as ensure the continuous growth of new bone. In this study, we used a finite element analysis (FEA) to explore the biomechanical distribution and observed new bone regeneration in clinical practice after implanting 3D printed prostheses for repairing metaphyseal bone defects. The results reflected that different fixation modes could result in diverse prosthesis mechanical conductions. If an intramedullary (IM) nail was applied, the stress mainly conducted equally along the nail instead of bone and prosthesis structure. While the stress would transfer more to the lateral bone and prosthesis’s body when the printed wing and screws are selected to accomplish fixation. All these fixation modes could guarantee the initial and long-term stability of the implanted prosthesis, but new bone regenerated with varying degrees under special biomechanical environments. The fixation mode of IM nail was more conducive to new bone regeneration and remodeling, which conformed to the Wolff’s law. Nevertheless, when the prosthesis was fixed by screws alone, no dense new callus could be observed. This fixation mode was optional for defects extremely close to the articular surface. In conclusion, our innovative study could provide valuable references for the fixation mode selection of 3D printed prosthesis to repair metaphyseal bone defect.

## 1 Introduction

The treatment of critical bone defects in extremities, especially those located at the metaphysis, remains a challenging issue for orthopedic surgeons. These defects are characterized by obvious morphological abnormality, as well as distinctive changes in microstructure and biomechanical distribution, considering there is the transition zone from cylindroid to irregular bone (Blokhuis, 2017). Metaphyseal bone defects of extremities can be caused by severe trauma, osteomyelitis, tumor and revision surgery (Mancuso et al., 2017; Lu et al., 2019; Benulic et al., 2020). Traditional therapeutic methods such as bone grafting, distraction osteogenesis, and induced-membrane technique cannot accomplish simultaneous reconstruction of both complete anatomical structure and stable biomechanical conduction to support early weight-bearing (Migliorini et al., 2021).

Applying 3D printed titanium alloy prostheses to repair critical metaphyseal bone defects is a feasible treatment option, whose major advantages include: 1) the shape and structure of a prosthesis can be customized based on the irregular defect outline; 2) the appropriate mechanical strength of titanium alloy can rebuild the local biomechanical stability and help patients perform weight-bearing and functional exercise; 3) it can stimulate the bone growth in contact with the prosthesis (Ji et al., 2020; Migliorini et al., 2021; Xu et al., 2021). Tetsworth et al. (2017) and Nwankwo et al. (2019) successfully applied 3D printed framed-structured scaffolds to reconstruct distal femoral and tibial metaphyseal defects caused by comminuted fractures. In addition, in our previous study, we reported the effective repair of distal femoral metaphyseal defects caused by osteomyelitis and nonunion using 3D printed porous prosthesis; the follow-up results showed stable prosthesis location and continuous bone growth (Hou et al., 2020). However, there is still a lack of systematic effect reports about the clinical application of 3D printed prostheses to reconstruct metaphyseal bone defects.

Durable prosthesis stability, new bone regeneration, and implant-bone interface fusion are desirable conditions after the 3D printed prosthesis implantation for bone defect repair. According to Wolff’s law, bone could do adaptive response to appropriate mechanical loading changes (Brand, 2010), that is to say, different internal fixation modes and biomechanical environments surrounding the prosthesis have a significant impact on the rate and direction of new bone growth. The mechanical mode of local fixation can be divided into the mode of interlocking intramedullary (IM) nail, the mode of the single prosthesis with screws, and the combination of the two (Tetsworth et al., 2017; Nwankwo et al., 2019). Too strong fixation can produce stress shielding and reduce the micromotion at the implant-bone interface, which in turn harms new bone regeneration, while too weak fixation leads to initial stability of prosthesis, which is not conducive to limb weight-bearing and may lead to implant failure. As for applying 3D prostheses to repair metaphyseal bone defects, no one so far has raised a unified reference standard about the optimal prosthesis design and fixation mode. Hence, there is still no definite conclusion on how to achieve the initial and long-term prosthesis stability as well as ensure the continuous growth of new bone.

Finite element analysis (FEA) has become an increasingly powerful approach for predicting the biomechanical behavior of the bone-implant interface and identifying areas of greater stress concentration (Campbell and Glüer, 2017). Once refined and validated with experimental data, FEA provides comprehensive and accurate datasets illustrating the physical response at all locations in a model. We carried out a FEA and prospective clinical study focusing on repairing metaphyseal defects in the present study. Following two research aspects were summarized and investigated in detail: 1) clarifying the stress distribution characteristics under different prosthesis fixation modes *via* 3D finite element models, and 2) investigating the rules of new bone regeneration related to different prosthesis fixation modes *via* the clinical study. We hypothesized that different fixation patterns could influence the local mechanical distribution and bone regeneration.

## 2 Materials and Methods

### 2.1 Design of a Customized Prosthesis

**FIGURE 1:**
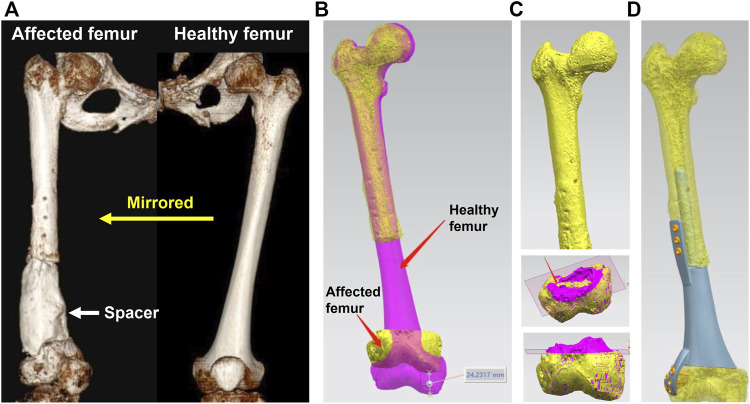
Design process of a 3D printed prosthesis. **(A)** 3D CT reconstruction of bilateral femurs, the bone defect was occupied by a cement spacer, the healthy femur was mirrored to the disease-affected side; **(B)** Overlapping femurs could reveal the size and shape of metaphyseal bone defect; **(C)** The overlapping 3D models could help to determine the osteotomy scope; **(D)** Simulation of the prosthesis insertion and internal fixation.

The design process of a 3D printed prosthesis is displayed in Figure 1. First, the computed tomography (CT) scan data of bilateral limbs were acquired and reconstructed using Mimics 19.0 software (Materialise, Belgium). Second, the healthy bone was mirrored to the disease-affected side, and the defect regions overlapped one another (automatically and manually). Third, the bone defect model obtained from Mimics was imported into Geomagic Studio 12 software (Geomagic, United States) for modification, after which personal prostheses with various shapes were designed according to specific defects and doctor’s requests. Fourth, the prosthesis insertion and fixation procedures were simulated *via* medical interaction platform; size, matching degree, and screw position was further evaluated. Any design that could not meet the surgical requirements was redesigned or modified and locally optimized.

### 2.2 Finite Element Analysis

FEA was used to accurately calculate the biomechanical distribution and concentration characteristics of bone and prosthesis (Yan et al., 2020; Yao et al., 2021). The metaphyseal bone defect of the distal femur was used as an example to conduct FEA models, and the results were applicable to all the long bones of extremities.

#### 2.2.1 Modeling of the Femur, Prosthesis, and Intramedullary Nail

For a better simulation of the real structure of the natural skeleton, cortical and cancellous bone were separately constructed based on the CT Hounsfield unit, and the thickness of cortical bone ranged from 1.4 to 8.8 mm. The elasticity modulus and Poisson’s ratio of cortical bone were set as 16,600 MPa and 0.3, and the elasticity modulus and Poisson’s ratio of cancellous bone were 8,000 MPa and 0.3. The 3D printed prosthesis was defined as porous titanium alloy structure with the elasticity of 3,000 MPa. The IM nail was defined as a cylinder, whose length and diameter were 400 and 11 mm respectively. Due to the IM nail was a solid titanium alloy model, its elasticity modulus and Poisson’s ratio were set as 110,000 MPa and 0.3. Surface smoothing and tanglesome point removal were performed using UG 12.0 software (Siemens PLM Software, United States), after which an STP file was acquired. After that, the STP file was imported into HyperMesh 14.0 software (Altair, United States) for geometric cleaning.

#### 2.2.2 Mesh Division

Since the bone-prosthesis interface experiences large deformations under load, it was necessary to mesh them into small elements. The mesh types included tetrahedral and shell elements, with the size of 2 mm. The tetrahedron simulated cancellous bone, and the shell unit simulated cortical bone.

#### 2.2.3 Bone-Prosthesis Contact Setting and Stress Loading

Interaction between the bone and prosthesis is complex and requires the definition of contact conditions. All contacts between fracture fragments and implants were modelled as frictional contacts, except the interfaces between threaded parts of the locking screw heads and plate holes, which were modelled as bonded contacts. The friction coefficients for the frictional contacts were set at 0.6 for bone-prosthesis interfaces. In applying stress, the proximal femur was secured, and a 700 N pressure was applied along the force line at the articular surface of the distal femur. The maximum von Mises and distribution were used to evaluate mechanical performance.

### 2.3 Clinical Study

This clinical study was approved by the Medical Science Research Ethics Committee of Peking University Third Hospital (No. M2018174), and informed consent was obtained from patients and family members. Besides, we have finished the clinical trials registry in the United States National Library of Medicine.

We prospectively enrolled patients with metaphyseal bone defects of extremities. The corresponding prosthesis type and internal fixation mode were selected according to different scopes and shapes of defects. The inclusion criteria included: 1) metaphyseal defects caused by osteomyelitis and nonunion; 2) the length of the bone defect was more than 6 cm; 3) patients aging from 18 to 70 years old. The exclusion criteria included: 1) tumor-related bone defects; 2) patients with articular surface defect; 3) systemic diseases contraindicating the use of artificial prosthesis; 4) patients unable to undergo the entire treatment process and follow-up. Patients’ clinical information were collected in detail, including age, gender, defect cause, defect location, defect length, fixation mode of the prosthesis, the evaluation scores of patients’ extremity function and life state.

The whole surgical process consisted of three main steps. First, the affected soft and bone tissues were debrided and cut thoroughly, and the bone defect was occupied by a polymethyl methacrylate (PMMA) spacer. Second, when the infection was completely contained, the critical metaphyseal defect was repaired using a 3D printed porous Ti6Al4V prosthesis. Finally, the interlocking IM nail or screws were used to enhance fixation according to specific situations.

Postoperatively, patients were encouraged to carry out early weight-bearing and functional exercises. The whole rehabilitation process was guided by professional specialists, including joint movement, anti-resistance and compression training, balance exercise and so on. Once the pain degree became significantly reduced after activity, and no loosening or breakage of implants were confirmed by postoperative X-ray, patients would be allowed to restore full limb weight-bearing gradually.

During the postoperative follow-up, routine X-rays and CT were utilized to assess implant stability and bone growth. Also, all complications were recorded. Patients’ upper and lower extremity functions were quantificationally evaluated by the Disability of Arm Shoulder and Hand (DASH) and Lower Extremity Functional Scale (LEFS), respectively. The state of patients’ daily life was evaluated *via* the SF-36 questionnaire.

### 2.4 Statistical Analysis

SPSS 22.0 software was used for statistical analysis. The Chi-square test was used to compare the excellent and good rate of extremity function scores among different groups. A F-test was used to compare the difference in SF-36 scores. *p* < 0.05 was considered to be statistically significant.

## 3 Results

### 3.1 Different Fixation Modes of Prostheses

**FIGURE 2:**
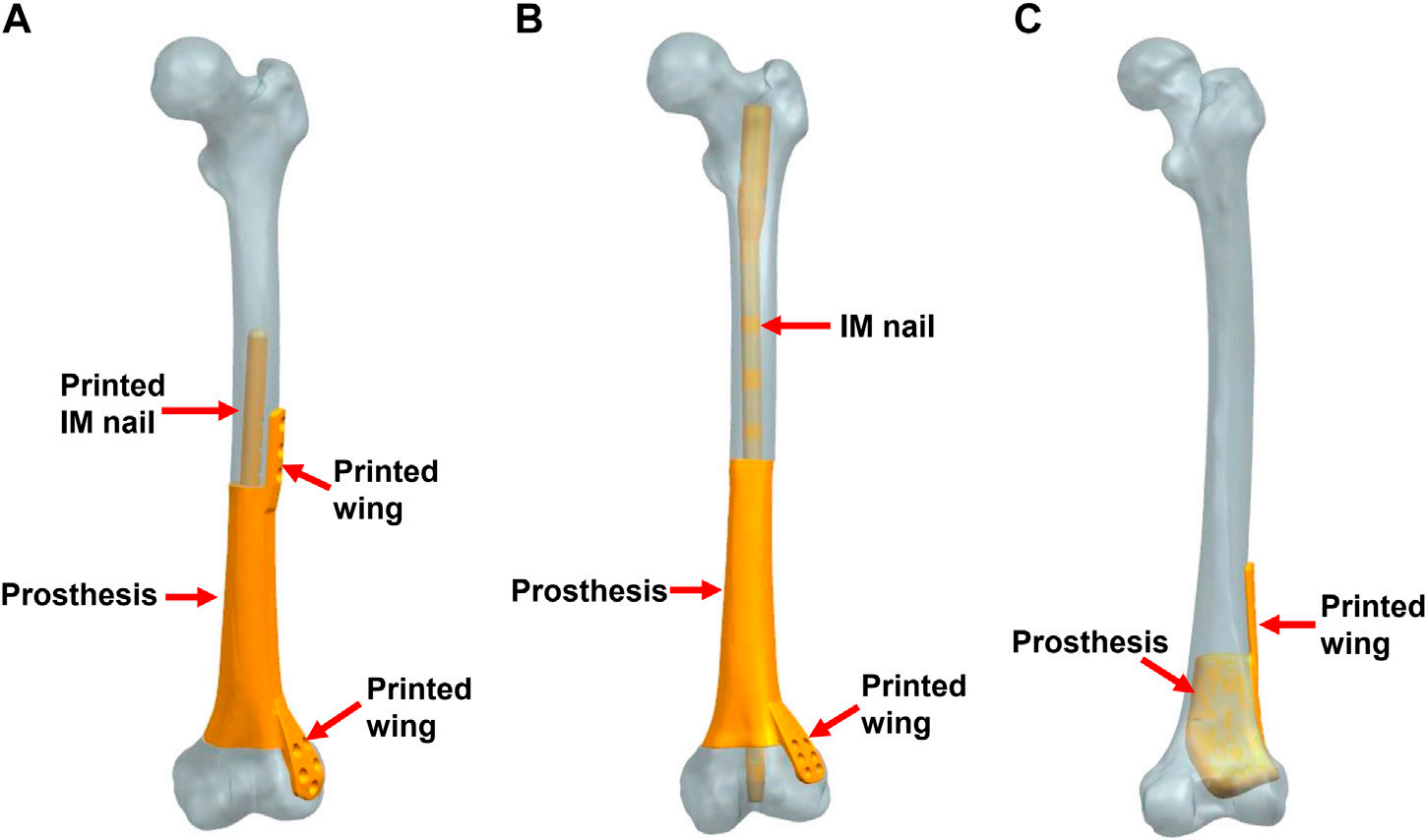
Three fixation modes of different prostheses (IM: intramedullary). **(A)** Mode I, the integral prosthesis with printed IM nail and wings; **(B)** Mode II, the integral prosthesis with one printed wing; **(C)** Mode III, the integral prosthesis with one printed wing.

A total of three modes of prosthesis fixation were accomplished. As shown in Figure 2, Mode I indicated the integral prosthesis with printed IM nails and wings (Figure 2A), which can be fixed by screws alone. Mode II indicated the integral prosthesis with one printed wing (Figure 2B), which can be fixed by interlocking IM nails and screws. There were two main differences between Mode I and Mode II: 1) the integratedly printed IM nail in Mode I could offer stabler prosthesis fixation strength compared with the additionally inserted IM nail in Mode II; 2) the proximal end of the prosthesis had integrally printed lateral wing, which would lead to less local relative micromotion at the bone-prosthesis interface after being fixed by screws. The different biomechanical environments around the proximal prosthesis might influence new bone regeneration. Mode III indicated the integral prosthesis with one printed wing (Figure 2C), which can be fixed by screws. The first two fixation modes of prostheses are suitable for most metaphyseal defects, and the third fixation mode of the prosthesis is only suitable for the partial metaphyseal defects, in which the bony structure on one side is intact and continuous.

### 3.2 Von Mises Stress Distribution Displayed by Finite Element Analysis

In FEA results, a qualitative and quantitative analysis was performed based on progressive visual color scales ranging from blue to red, which corresponded to the weakest and the strongest stress intensity, respectively.

**FIGURE 3:**
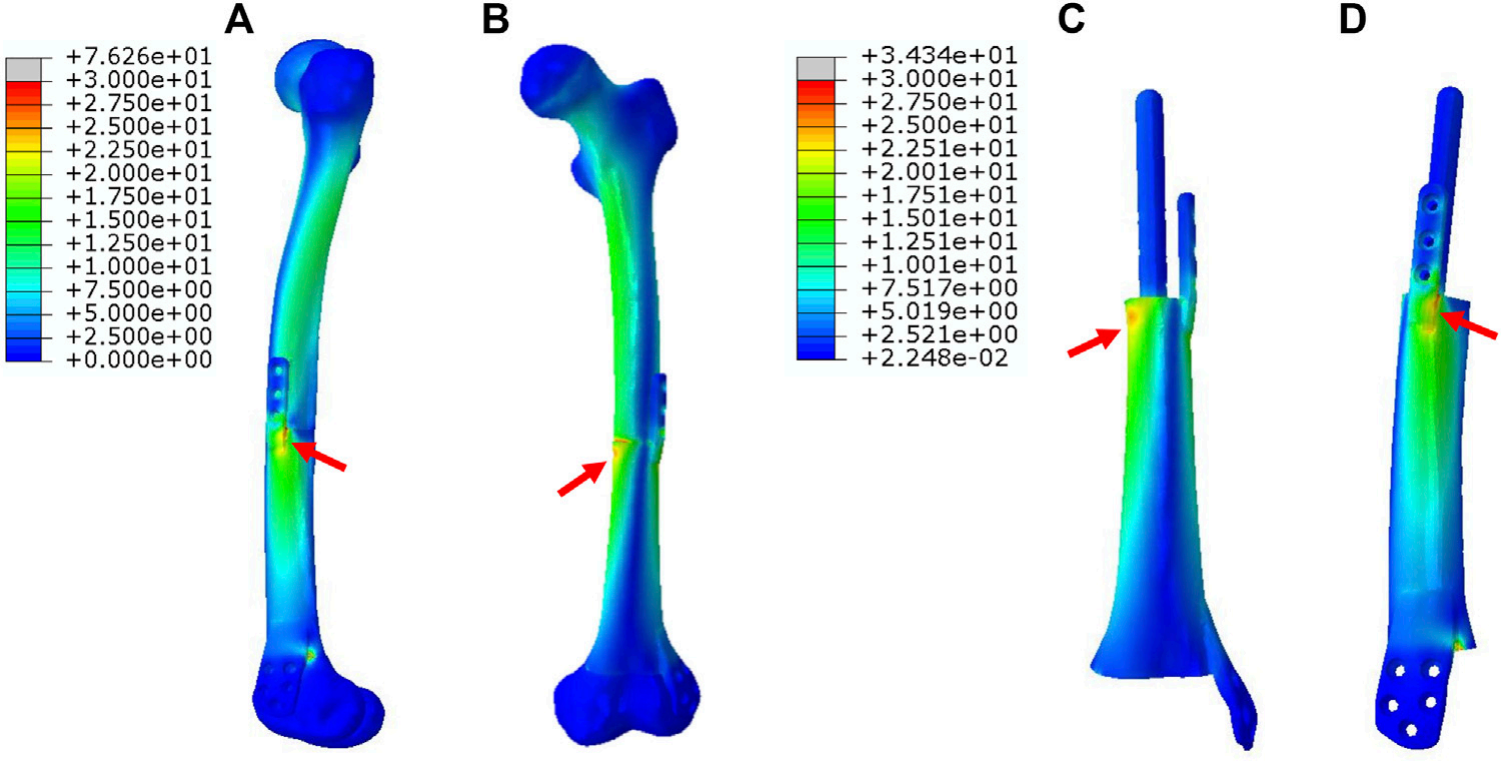
Von Mises stress distribution in Mode I fixation pattern, the red arrows direct the maximum stress concentration.

In Figure 3, for Mode I, the stress was evenly conducted along the inner and outer sides of both femur and prosthesis, with the maximum stress located at the proximal prosthesis end as directed by red arrows. The stress distributed on the printed IM nail was relatively weak. The proximal printed wing shared stronger stress than the distal wing.

**FIGURE 4:**
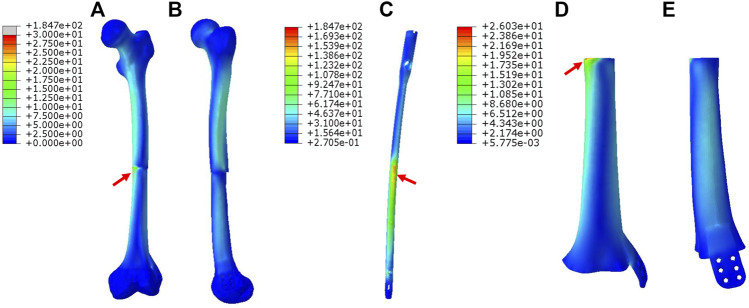
Von Mises stress distribution in Mode II fixation pattern, the red arrows direct the maximum stress concentration.

In Figure 4, for Mode II, the stress was mainly conducted along the femurs and IM nails. The maximum stress was concentrated at the middle of the IM nail and the proximal end of the prosthesis as directed by red arrows. The printed wing shared less stress.

**FIGURE 5:**
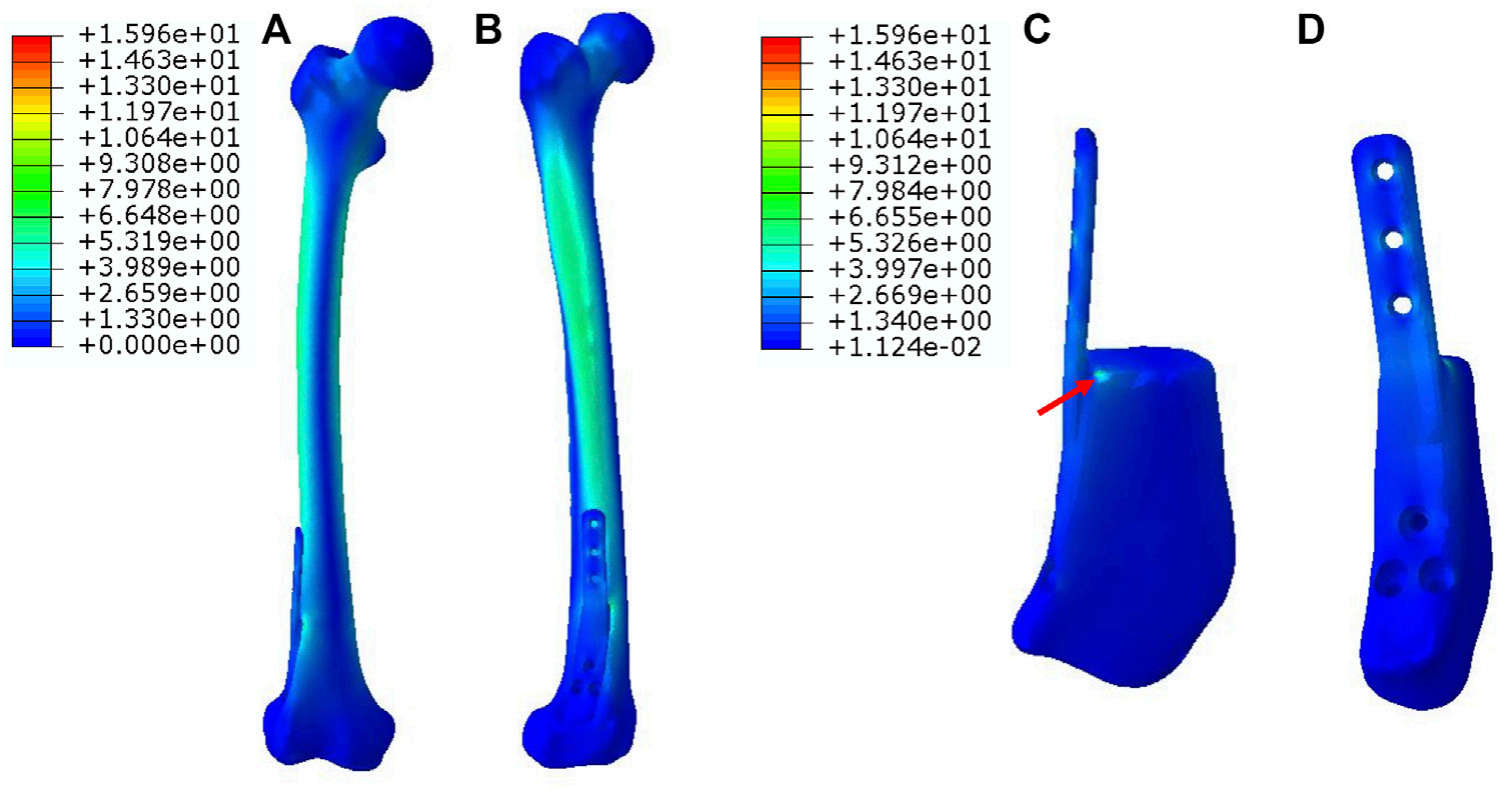
Von Mises stress distribution in Mode III fixation pattern, the red arrow directs the maximum stress concentration.

In Figure 5, for Mode III, the stress was mostly conducted along with the femur structure uniformly. The stress distributed on the overall prosthesis was relatively weak. The maximum stress of the prosthesis is concentrated on the proximal end as directed by red arrows.

**FIGURE 6:**
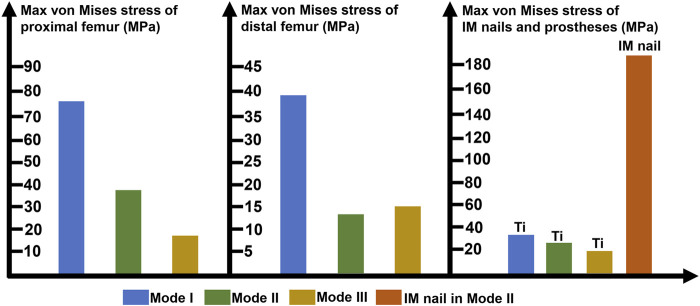
Diagram of the maximum von Mises stress of proximal femur, distal femur, prostheses and intramedullary (IM) nail (Ti: 3D printed Ti6Al4V prosthesis).

The maximum von Mises stress intensity of the proximal femur, distal femur, and implants (IM nail and prosthesis) are presented in Figure 6. The maximum von Mises values of proximal and distal femur were observed in Mode I (76.1 and 38.8 MPa), and their minimum values in Mode III and Mode II, respectively (17.2 and 16.4 MPa). The interlocking IM nails were applied in Mode II, which decreased the maximum stress of the femur. However, the von Mises stress on the IM nail was significantly larger than the prosthesis, which was 184.7 MPa.

### 3.3 Prosthesis’s Stability and Bone Regeneration Under Different Fixation Modes.

**FIGURE 7:**
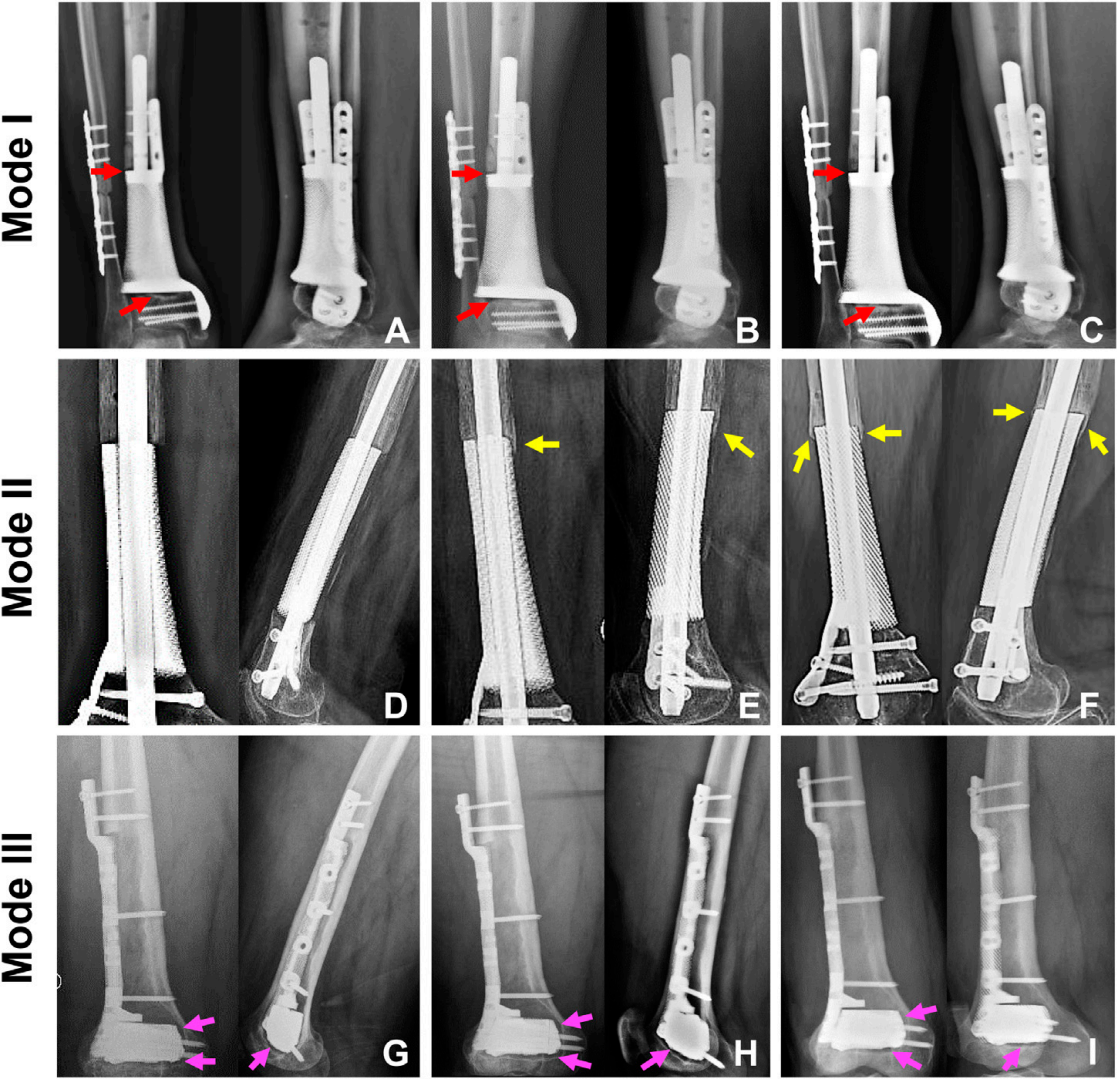
The state of prosthesis’s stability and new bone regeneration in three groups. For Mode I, the typical patient was a 65-year-old male who suffered distal tibial metaphyseal defect due to osteomyelitis. Along with his postoperative X-rays at 1 **(A)**, 7 **(B)**, and 22 **(C)** months, we could observe the stable location of both prosthesis and screws. The low-density gap at bone-prosthesis interface gradually blurred but not disappeared as directed by the red arrows. For Mode II, the typical patient was a 67-year-old female who suffered distal femoral metaphyseal defect due to osteomyelitis. Along with her postoperative X-rays at 1 **(D)**, 9 **(E)** and 25 **(F)** months, we could observe the stable location of prosthesis, nail and screws. Besides, new bone continuously regenerated around the prosthesis as directed by the yellow arrows. For Mode III, the typical patient was a 26-year-old male who suffered distal femoral partial metaphyseal defect due to osteomyelitis. Along with his postoperative X-rays at 1 **(G)**, 5 **(H)** and 14 **(I)** months, we could observe the stable location of both prosthesis and screws. The low-density gap at bone-prosthesis interface existed persistently as directed by the pink arrows.

We treated 16 patients, eight males and eight females, with critical metaphyseal bone defects of extremities by implanting 3D printed porous prostheses. Their average age was 54.4 years old. The enrolled patients were divided into three groups according to different prosthesis fixation modes: group 1: five patients underwent Mode I; group 2: six patients underwent Mode II; group 3: five patients underwent Mode III. Their clinical information were presented in Table 1. As shown in Figure 7, postoperative serial X-rays showed that the prostheses, nails, and screws were stable in three groups, and no obvious loosening, subsidence, or breakage occurred. Yet, the new bone regeneration was not the same in all groups. For Mode II, continuous bone regeneration could be observed. As directed by yellow arrows, new bone mainly grew from the proximal end of the bone defect and crept on the surface of the prosthesis, whereas, for both Mode I and III, the new bone did not grow densely. As directed by red and pink arrows, the low-density gaps between the bone and prosthesis were gradually blurred but did not disappear. There was no obvious new bone growing around the prosthesis.

**TABLE 1:** The clinical information of the enrolled patients.

	Age/Gender	Characteristics of bone defects			Fixation mode	F/U (mths)	Scores at the last follow-up	
		Cause	Location	Length (cm)			LEFS/DASH	SF-36
1	64/F	osteomyelitis	distal femur	17.2	II	40	51	57.6
2	61/M	aseptic nonunion	distal femur	14.1	II	38	57	48.1
3	69/F	aseptic nonunion	distal femur	9.5	II	30	46	56.5
4	67/F	osteomyelitis	distal femur	13.3	II	26	51	51.1
5	41/M	osteomyelitis	distal femur	14.2	I	18	47	63.6
6	26/M	osteomyelitis	distal femur	17.8	III	16	61	69.6
7	36/M	aseptic nonunion	distal femur	17.3	II	13	56	46.0
8	42/F	aseptic nonunion	distal femur	14.4	II	26	48	55.7
9	67/F	osteomyelitis	distal femur	10.5	III	14	56	55.1
10	46/F	osteomyelitis	distal femur	14.2	I	10	44	53.4
11	65/M	osteomyelitis	distal tibia	9.7	I	23	55	64.5
12	68/M	osteomyelitis	proximal tibia	14.2	I	23	56	53.2
13	69/M	osteomyelitis	proximal tibia	6.7	III	14	55	57.2
14	54/M	osteomyelitis	distal tibia	6.8	III	10	58	54.2
15	53/F	aseptic nonunion	distal humerus	9.5	I	25	26	64.5
16	43/F	osteomyelitis	proximal humerus	7.1	III	14	19	66.2

F, female; M: male; Pre-Op, Preoperative operative; F/U, Follow-up; LEFS, lower extremity functional scale; DASH, disability of arm shoulder and hand.

### 3.4 Evaluation of Patients’ Extremity Function and Life State

At the last follow-up, the excellent and good rate of extremity function scores (LEFS or DASH) did not show significant difference between Mode I and Mode II groups (*p* = 0.819). While the rate of Mode III group was significantly higher than other two groups (*p* = 0.038 and 0.022, respectively). With regard to the comparison of SF-36 scores at the time of the last follow-up, no significant difference was found among the three groups (*p* = 0.076).

## 4 Discussion

Due to special characteristics such as customized morphology and appropriate mechanical strength (Tshephe et al., 2022), 3D printed titanium alloy prostheses have been widely applied to repair bone defects in extremities. In this study, we investigated the biomechanical distributions of different prosthesis fixation modes combined with finite element 3D simulated models and observed the treatment effects of 3D printed Ti6Al4V prostheses in critical metaphyseal bone defects. The reported results could represent a valuable reference for orthopedic surgeons to make clinical decision-making.

Different prosthesis fixation leads to different local biomechanical environments. As shown in Figure 2, the three fixation modes of prostheses for repairing metaphyseal bone defects proposed in this study were classified into two categories based on whether the interlocking IM nail was applied. On the one hand, when the prosthesis was fixed by IM nails and screws, the stress under such conditions could be transmitted along with the IM nail, whose main curvature is close to the normal force line and the interlocking screws have a good anti-rotation and shear resistance function. Besides, studies have demonstrated that when applying a vertical load to simulate the early postoperative internal fixation load-bearing, the stress is mainly concentrated in the middle of the IM nail (Chen et al., 2018; Wang et al., 2020). Wu et al. (2022) applied the 3D printed polyether ether ketone prosthesis to repair the femoral shaft defect and observed middle-concentrated stress of the IM nail. As shown by the FEA results in the present study, when the interlocking IM nail was applied to stabilize prostheses for metaphyseal bone defects, the stress was mainly distributed along the IM nail (the maximum stress concentration occurred at the middle of the nail) (Figure 4), which is consistent with previous studies. In addition, the screw fixation between the lateral wing and distal bone mass could increase the local stability between the prosthesis and the bone, promoting the micromotion at the local bone-prosthesis interface. On the other hand, for metaphyseal bone defects located close to the articular surface and possessing irregular shapes, there is little residual bone near the articular surface, and the interlocking fixation is difficult to perform. Hence, the prosthesis was printed with integrally lateral wings and/or nails and was only fixed by screws. Under these circumstances (Figure 3), the stress shared by the printed nail was relatively weak and was mainly conducted along the bone structure and prosthesis body. At the same time, the function of the printed lateral wing was similar to a plate, which could provide eccentric fixation force and result in lateral stress concentration. As for the fixation pattern in Mode III (Figure 5), the partially intact bone structure became the main pathway of stress conduction; the stress shared by the prosthesis body and printed wing were both relatively weak.

Sustained prosthesis stability, which can be divided into primary and secondary stability, is the main guarantee for patients to safely carry out limb weight-bearing. The primary stability refers to the period after surgical insertion, before the healing progress. After implanting the prosthesis inside a defective cavity, the nail and screws can provide necessary mechanical fixation, and the frictional properties at the bone-implant interface can sustain the shear load and ensure proper implant stability. In addition, the nail and screws can also provide necessary mechanical fixation. Insufficient primary stability tends to lead to excessive interfacial micromotion following surgery, which may imply a higher occurrence of migration and implant failure. Besides, the low fixation strength brings no additional benefit to the healing process and can actually promote the growth of fibrous tissue instead of bone. From another aspect, the primary stability and fixation strength should not be too high since excessive stress levels may lead to bone necrosis (Campbell and Glüer, 2017). Therefore, a combination of IM nail and lateral screws was chosen for clinical application to fix the metaphyseal prostheses in this study, avoiding the too strong or too weak fixation state and meeting the mechanical environment requirements for primary stability and new bone regeneration. The secondary stability occurs in the stage of bone regeneration and remodeling. During bone healing, newly formed bone is produced to fill the gap between host bone tissue and prosthesis surface. Several weeks or months after the implantation surgery, newly formed bone tissue is progressively replaced by mature bone tissue, thus promoting mechanical interlocking. As shown in Figures 7D–F, the new bone gradually surrounded the prosthesis from the proximal end. Bone-prosthesis interface is suggested to be the weakest domain in the bone-implant complex (Gao et al., 2019). Osseointegration, which was first defined by Brånemark et al. (1977), helps integrate and stabilize the prosthesis with the surrounding bone and has an important role in strengthening the bone-prosthesis interface. As shown in Figure 7, the three fixation modes that were applied in the present study all achieved good early and medium-term prosthesis stability.

After the prosthesis is implanted into the bone defect, the regeneration process of new bone is mainly achieved with “secondary bone repair” (Roberts et al., 2018). During this process, the growth and remodeling of new bone follow Wolff’s law, illustrated by the increased bone resorption associated with immobilization or microgravity and increased bone formation in response to mechanical load. In this study, different fixation modes led to different subsequent stress distributions and affected the regeneration of new bone. As shown in Figures 7A–C and Figures 7G–I, the fixation of the prosthesis with screws alone resulted in concentrated biomechanical conduction between bone and prosthesis interface, which induced the new bone to grow gradually at the bone-prosthesis gap instead of growing on the surface of prostheses. At the same time, the application of IM nail was more conducive to the surrounding new bone regeneration (Figures 7D–F). Under this circumstance, stress is equably distributed along with the IM nail and bone-prosthesis interface, resulting in new bone growth from the end of the bone defect, continuously surrounding the prosthesis. Generally, an adaptation of bone structure to the mechanical environment consists of several mechanisms. The trabecular bone exhibits a phenomenon in which the apparent bone density and orientation characteristics change according to the stress magnitudes as well as the principal directions. The structure change couples the bone resorption by osteoclastic cells and bone formation by osteoblastic cells. The various cells contributing to the remodeling detect mechanical stimuli and transduce them to biochemical signals (Gao et al., 2019). This complex process can be changed according to the local mechanical environments. In this way, bone remodeling based on mechanical stimulus at the cellular level leads to structural and functional adaptive changes corresponding to the load direction and magnitude, aiming to achieve a uniform mechanical stimulus state.

The limb function of all patients could meet the weight-bearing requirements of daily life at the last follow-up, meaning all patients benefited from the advantages of customized titanium alloy prostheses and stable internal fixation options. Patients in Mode III group had a significantly higher rate of excellent or good limb function scores, which was associated with the initially smaller extent of bone loss in these patients. Besides, at the last follow-up, there was no significant difference in the SF-36 scores among the three groups, proving that applying 3D printed prostheses could achieve similarly good clinical curative effects in patients with metaphyseal bone defects of extremities.

The main limitation of this study was the number of included patients, which was relatively small. In our future work, we plan to include more cases and conduct a wider multicenter clinical study in view of applying 3D printed prostheses to repair metaphyseal bone defects, which will be more conducive to the systematic investigation on clinical efficacy and new bone growth.

## 5 Conclusion

The treatment of metaphyseal bone defects is a great challenge in clinical practice. Applying 3D printed prostheses to repair metaphyseal bone defects of extremities can result in good recovery of limb function, thus allowing patients to normally engage in their daily activities. Yet, different fixation patterns can lead to different biomechanical distributions. The use of IM nails for prosthesis fixation is more conducive to equal conduction of stress and new bone regeneration. Besides, the fixation mode in which the prosthesis was only fixed by screws was also optional for defects extremely close to the articular surface.

## Data Availability

The original contributions presented in the study are included in the article/supplementary material, further inquiries can be directed to the corresponding authors.
